# Vaccination

**Published:** 1901-12

**Authors:** F. E. Daniel

**Affiliations:** Austin, Texas, in “The Current Issue”


### Vaccination.

F. E. DANIEL, M. D., AUSTIN, TEXAS, IN "THE CURRENT ISSUE."

The existence of a widespread epidemic of smallpox in Texas and many other States, and the inability of sanitary authorities to control it, makes the discussion of vaccination appropriate at this time. Especially is it so, because of the ignorance of the subject on the part of the people at large, and the consequent prejudice against vaccination, the only means known to science whereby the disease may be arrested. This prejudice has been fostered, and a strong opposition to the use of the measure aroused, partly by certain persons who are asserting and publishing statements to the effect that smallpox can be prevented by simpler and less dangerous (?) means: for instance, by swallowing little sugar-of-milk pellets dipped in an infinitesimally attenuated solution of vaccine lymph. The absurdity of such a claim is not apparent to the people; but every medical man knows that one may swallow an entire vaccine scab, and acquire no immunity thereby; it is simply digested, as any other animal substance is, losing its distinctive character. Snake venom, even, can be swallowed with impunity, whereas if introduced into the system through the skin, its poisonous effects are at once manifested. Others rely upon prayer and faith. To thus trade upon the ignorance and credulity of a trusting clientelle,—as certain persons are doing, lulling them to repose, in a false sense of security, is little, if anything, short of criminal. The opposition to vaccination grows partly, too, out of what those who oppose it have seen, or heard, of evil results from vaccination under the old methods. Many persons will give as a reason for objecting to vaccination, that they have seen, or known of, badly inflamed arms, great sores, fever and headache, etc., resulting from vaccination. All that is now changed. The days of sore arms and blood poisoning are past, and modern methods of gathering and preserving the lymph, and of performing the operation, have robbed the process of its dangers, and reduced it to a minimum of inconvenience.

It can readily be understood that under the old methods,—when less care was taken in procuring and protecting the lymph than now; when the operation of vaccination was done without the precautions of antisepsis, frequently by a layman, some member of the family, or by some dirty or slovenly "doctor," perhaps with a lancet that had been used for other purposes, or a rusty needle, or with a dirty pocket-knife,—something besides vaccine lymph was introduced into the arm; some septic matter from the atmosphere, or the lancet or knife,—and *septicaemia* (blood poisoning) often resulted, causing much inflammation, soreness, fever, aching, and even ulcers which were slow to heal. Especially was this apt to occur when, as was the case during the war, and, until pure virus was more easily obtained, "humanized virus" was used; that is, a virus that had passed through the system of some one or more persons,—the scab from the arm of the first person being used to inoculate the others. In such cases it can readily be seen that there was not only danger of blood poisoning, from the introduction into the arm of dried blood, or shreds of fibrin (dead animal matter), or *pus*,—but of inoculating the germs of some disease, as syphilis, for instance, in case the first party vaccinated should be the subject of such disease. During the war, such inoculation as a sequence of vaccination was not uncommon. Hence, in light of such experiences, the objection to vaccination is not to be wondered at. But, as said before, all that is now changed; and under modern methods vaccination is not only not dangerous, but is perfectly harmless, and causes only the slightest inconvenience. Moreover, it is almost *absolutely protective*, and is the *only means whereby smallpox will ever be exterminated.*

The people should be enlightened on the subject; they should be shown the results throughout the world that have been observed and gathered and tabulated, results upon which the above assertion is based. They should be made to see and intelligently understand that the only safety to themselves, their families, and the whole community lies in vaccination, and, if necessary, every few years—if exposed to infection—revaccination. For let it be understood that vaccination, when properly done, with fresh and pure virus, does not always give immunity *for life*. It may be, and most frequently is, entirely protective; but sometimes it protects only in part, and when a person who has been successfully vaccinated contracts the disease, as is sometimes, but very rarely, the case, he has a modified smallpox,—*varioloid*. I mean by that: The experience of the world is that to insure the fullest measure of protection one should be vaccinated in infancy, and again in from seven to nine years. If repeatedly exposed to the infection of smallpox, it is best to still again be vaccinated. The above method is, I think, the rule in Germany and France. Still, persons who have two, three, even four scars (not always indicative of successful vaccination), occasionally, very rarely indeed, have varioloid, but it is seldom fatal.

The lymph used in the present day is taken from healthy young heifers, under the strictest sanitary and antiseptic precautions, under the supervision of capable and conscientious physicians. There is an establishment where it is done under government supervision; and from all the great vaccine establishments the lymph is sent out under guarantee of purity, with date of the extraction stamped on the package, so that one may know the age; and with strict injunctions to return it (if the "dry" kind, on "points") if not used, and exchange it for a fresher package. It is put up in sterilized packages, or "glycerinated"; in fact, every precaution is taken to insure its purity and to preserve it from contamination.

The operation of vaccination should be performed by a physician, and if he be a capable man, he will take the same precautions that he would do in any other surgical operation, to insure *asepsis*. That is, his hands will be cleansed; his lancet, if not a new one, should be held in the flame of an alcohol lamp or dipped in boiling water. The spot selected for the operation should be thoroughly cleansed, preferably with ether, after washing and drying, or with ether-soap. No blood should be drawn, but the arm should be scratched until there is a slight oozing of lymph, and the vaccine should be spread on and rubbed in. A protective shield, made for the purpose, and to be had from all depots of vaccine lymph, should be worn until the pustule "matures," and the "scab" dries up and falls off. It should never be forcibly removed or rubbed off. When the operation is done as described, and with fresh and pure lymph, it is almost sure to "take," and will cause no inflammation or other distressing symptoms. In a few days a small vesicle filled with a clear fluid (lymph) will appear, surrounded by a small area of infiltrated skin—an areola—which should not be larger than a ten cent piece. At the end of eight or ten days the vesicle has "matured," its contents having become first milky, then purulent, a pustule is formed, and then begins the decline, which results in the formation of a dried, indented ("umbillicated"), scab, which will fall off later, and will leave a scar, "pitted" with what is known as "strawberry" pits or indentations.

Were the people enlightened upon the subject, and assured of the fact, for it is a fact, well established and demonstrated, that vaccination, when properly done, not only occasions no suffering or inconvenience and involves no danger whatever, but will afford almost absolute safety in the midst of this dreadful disease, all rational people would hail it as a boon and blessing. Amongst the unenlightened, the people should be persuaded, for their own safety and the safety of the community, to submit to vaccination, and the densely ignorant who cannot be convinced should be subjected to the process as a means of public safety. The State should pass a law which shall be practically compulsory. Rational people will need no compulsion. In some such measure lies the only hope of exterminating the pest. In this question is involved a problem upon the solution of which depend thousands of lives and millions of money. Vaccination, as has been demonstrated in England and in Germany and in France, offers the only solution. Quarantining against it, and quarantining those down with the disease, and those who have been exposed to it, is a failure, as has been clearly shown, not only in Texas, but in other American States. Where will it end? It will not end until thousands of lives and untold sums of money have been wasted; until the entire unvaccinated population have had the disease, and pock-marks will have become a distinguishing feature of every other individual one meets, as it is among the masses in Mexico. Texas should profit by the lesson learned in other countries and States, and follow the examples set by them in resorting to the only effectual means of arresting the disease. As before said, the intelligent class will need no compulsion; they will not need the services of any health officer, but will have their physician to perform the operation. I will give presently an illustration of what I mean by the "lessons learned" and the "example set" by more enlightened people. Before doing so, I wish to call attention here to a danger which, if not guarded against by timely measures, will touch not only the public health, but the pocket-book of State and people in a quarter very dear to their hearts.

Cotton is the greatest source of wealth to Texas. Cotton is a dangerous carrier of the infection, and as negroes and Mexicans with smallpox were engaged in cotton picking the past fall, and others just recovered, and before the scabs of the loathsome disease had been shed, there is no knowing where or how far the infection has been disseminated. Texas need not be surprised some morning to wake up and find Texas cotton quarantined in all parts of the civilized world; and cotton raising countries would want no better pretext, and could have none, than the danger of introducing the disease into that territory.

In 1870-1, during the Franco-Prussian war, 43,000 German *soldiers* died of smallpox, and 23,000 French. In 1874, Germany enforced vaccination throughout the empire, and the annual death rate from smallpox amongst 70,000,000 people was reduced to 116. (See *Reports United States Marine Hospital Service,* January 6, 1899.) England exterminated the disease during the time the compulsory vaccination act was in force, and I have the authority of Andrew Dickson White, United States Embassador to Russia, and long time president (and joint founder) of Cornell University, for the statement that in 1890 only one death from smallpox occurred in London. (See President White's great book: *"Warfare of Science and Theology."*) To come nearer home: I have the authority of our own State Health Officer, Dr. Blunt (and it is a matter of record as well), for saying that, at Laredo, two years ago, 1200 cases of smallpox occurred amongst the unvaccinated Mexicans, of whom over twenty-six per cent. died (some 320); while in the 2200 white population, all of whom but *one* were vaccinated (an old lady who begged to be not vaccinated), there occurred twelve cases (varioloid) and one death—the old lady referred to.

If stronger evidence of the protective value of good vaccine were needed, it is to be found in the fact that it will protect a baby born of a mother suffering with smallpox at the time of its birth; and will protect those who have been in direct contact with the disease at its height, if done within three, four or five days after the exposure.

In *"A Statistical Record of 5000 Cases of Smallpox"* observed by him, Professor W. M. Welch, of the University of Pennsylvania, and Surgeon of the Hospitals for Infectious Diseases in Philadelphia, the highest authority in America on smallpox and vaccination, referring to a statistical table (which I regret I cannot introduce here) says:

"It is impossible to illustrate in this table the very striking and conclusive evidence of the protective power of vaccination that frequently came under my notice while observing these five thousand cases of smallpox—such, for instance, as witnessing, on the one hand, an unvaccinated person suffering from the confluent form of the disease, loathsome and offensive, with the final issue for several days uncertain, and on the other hand a vaccinated person undergoing a modified form of the disease, so mild and innocent in its character as not to excite any apprehension for the safety of the patient. In the former case, if recovery took place, the individual was left disfigured for life, while in the latter, after a few months had passed, there was but little, if anything, in the appearance of the individual to indicate that he had ever suffered from the disease at all.

"Also, I have seen over and over again entire families brought into the hospital when all the unvaccinated children have been suffering from smallpox and the vaccinated children unaffected; have seen the former perish and the latter remain exempt from the disease, although living, eating, and sleeping in the infected atmosphere for several weeks. But I have yet to see a single unvaccinated child escape the disease under similar circumstances. Furthermore, I have more than once seen a vaccinated infant draw its daily supply of nourishment from a mother suffering from varioloid, and the infant remain as free from any symptom of the disease as if the infection were a thousand miles away and the food were received from a most wholesome source. All this is evidence of the prophylactic power of vaccination that cannot be shown in mortality tables."

The history of vaccination, in all countries, is an unbroken record of success in arresting the spread of and exterminating smallpox. Hundreds of instances could be given similar to the above, did space permit. Especial reference is made to Loomis' *System of Medicine,* to Osler's *Practice of Medicine,* and to Buck's *Reference Handbook of the Medical Sciences,* Vol 7, for statistics of vaccination in America. Several of the States in America have compulsory vaccination laws. In many States there are laws that are practically compulsory, if not literally so. For instance, in many States no child is permitted to enter any school without a certificate of successful vaccination. In some the prohibition extends to all industries where operatives are employed; mills, factories, etc. I am under the impression that the United States immigration laws prohibit the landing of any foreigner without evidences of successful vaccination. Were such evidences made the *sine qua non* to getting employment (especially cotton picking), or of sending a child to school, in a very short time the good effect of the reform would be felt, and we would have, practically, "compulsory vaccination."

